# Complete pathological response to olaparib and bevacizumab in advanced cervical cancer following chemoradiation in a *BRCA1* mutation carrier: a case report

**DOI:** 10.1186/s13256-021-02767-9

**Published:** 2021-04-23

**Authors:** Rosa Montero-Macias, Meriem Koual, Céline Crespel, Marie Aude Le Frére-Belda, Hélène Blons Hélène, Huyen-Thu Nguyen-Xuan, Simon Garinet, Géraldine Perkins, Vincent Balay, Catherine Durdux, Marie Florin, Hélène Péré, Anne-Sophie Bats

**Affiliations:** 1 Department of Gynaecologic and Breast Oncological Surgery, European Georges-Pompidou Hospital, APHP. Centre, 20, rue Leblanc, 75908 Paris Cedex 15, France; 2 Faculty of Medicine, Paris University, Paris, France; 3 Centre Universitaire des Saints-Pères, INSERM UMR-S 1124, Université de Paris, Paris, France; 4 Department of Medical Oncology, European Georges-Pompidou Hospital, APHP. Centre, Paris, France; 5 Department of Pathology, European Georges-Pompidou Hospital, APHP. Centre, Paris, France; 6 Department of Biochemistry, European Georges-Pompidou Hospital, APHP. Centre, Paris, France; 7 Centre Universitaire des Saints-Pères, INSERM UMR-S 1147, Université de Paris, Paris, France; 8 Department of Biology, European Georges-Pompidou Hospital, APHP. Centre, Paris, France; 9 Department of Radiotherapy, European Georges-Pompidou Hospital, APHP. Centre, Paris, France; 10 Department of Radiology, European Georges-Pompidou Hospital, APHP. Centre, Paris, France; 11 Department of Virology, European Georges-Pompidou Hospital, APHP. Centre, Paris, France; 12 INSERM 970, Paris Centre de Recherche Cardiovasculaire (PARCC), European Georges-Pompidou Hospital, APHP. Centre, Paris, France

**Keywords:** Advanced cervical cancer, *BRCA1*, PARP inhibitor, Olaparib, Precision oncology

## Abstract

**Background:**

Homologous recombination deficiency is a marker of response to poly(ADP-ribose) polymerase inhibitors in different cancer types including ovary, prostate, and pancreatic cancer. To date, no report about poly(ADP-ribose) polymerase inhibitors has been published on cervical cancer.

**Case presentation:**

Here we present the case of a patient with cervical cancer treated in this setting. A 49-year-old woman diagnosed with International Federation of Obstetricians and Gynecologists stage 2018 IIIC2 locally advanced undifferentiated cervical cancer received first-line chemoradiotherapy followed by carboplatin, paclitaxel, and bevacizumab with partial response. Because of a family history of cancers, the patient was tested and found positive for a pathogenic *BRCA1* germline and somatic mutation, which motivated bevacizumab plus olaparib maintenance treatment. A simple hysterectomy was performed after 2 years stable disease; pathological report showed complete pathological response, and 12 months follow-up showed no recurrence.

**Conclusion:**

Poly(ADP-ribose) polymerase inhibitors could be an alternative maintenance treatment for patients with persistent advanced cervical cancer previously treated with platinum, especially when familial history of cancers is reported. Clinical trials using poly(ADP-ribose) polymerase inhibitors for advanced cervical cancer are warranted.

## Background

Cervical cancer (CC) is the fourth most frequently diagnosed cancer and the fourth leading cause of cancer death in women worldwide [1]. Squamous cell carcinomas is the most common histological type, and although the difference in survival outcomes with adenocarcinoma remains controversial, there are studies that have shown a significantly greater response to adjuvant treatment in squamous cell subtype compared with adenocarcinomas, as well as difference in immunological microenvironments and tumor escape mechanisms [2].

Thirteen percent of CC cases are diagnosed at an advanced stage, and even if patients are treated with curative intent, responses are of short duration and prognosis is very poor with recurrent and metastatic disease. Five-year survival rate in advanced stages is about 20–60% [3]. Treatment of locally advanced CC is based on chemoradiation, and recurrent, persistent, or metastatic disease is treated first-line with chemotherapy and bevacizumab [4, 5]. There are currently no clear recommendations for second-line treatment in these cases. A retrospective study showed that 70% of women treated for recurrent or metastatic CC with systemic therapy for recurrent or metastatic CC subsequently received second-line therapy with an overall response rate (ORR) of 13.2%, a median progression-free survival (PFS) of 3.2 months, and a median overall survival of 9.3 months [6]. Recent advances in molecular biology of cancer have led to the development of targeted therapies in gynecological cancers, including poly(ADP-ribose) polymerase inhibitors (PARPi) [7–9]. This novel class of anticancer drug is approved in various indications, such as high-grade serous ovarian cancer and metastatic breast cancer, and is under investigation in many other types of malignancies [10]. To date, no report about PARPi has been published on CC.

Here, we report the case of a 49-year-old *BRCA1* mutated woman diagnosed with advanced CC treated by chemoradiation, followed by the combination olaparib/bevacizumab with complete pathological response.

## Case presentation

A Caucasian 49-year-old patient was referred to our center in January 2016 with a uterine mass of 6 cm discovered during an ultrasound examination performed for lumbar pain. Clinical examination showed a friable suspect cervix with bilateral parametrial involvement. Pelvic magnetic resonance imaging (MRI) revealed a 91-mm cervical and uterine mass, with involvement of the uterine serosa, left distal parametrium, left pelvic wall, and left hydronephrosis. Imaging revealed close contact with the rectal wall and bladder trigone without transmural invasion and a suspicious left external iliac adenomegaly. Positron emission tomography/computed tomography (PET/CT) showed no evidence of paraaortic lymph node involvement or distant metastasis. Cervical biopsy found a poorly differentiated cervical carcinoma human papilloma virus (HPV) 16 positive.

A laparoscopic extraperitoneal paraaortic lymphadenectomy was performed in February 2016. Pathological report showed two nonmetastatic left external iliac nodes (2N-/2) and 23 paraaortic lymph nodes including five metastatic nodes (5N+/23): International Federation of Obstetricians and Gynecologists (FIGO) 2018 stage IIIC2.

The patient received two cycles of capecitabine/cisplatin and subsequent concurrent chemoradiation (64,8 Gy in 36 fractions in the pelvic area, 45 Gy in 25 fractions in iliac and paraaortic area, and 8 concurrent cycles of cisplatin). The treatment was completed in June 2016.

At 3 months follow-up (September 2016), MRI showed partial response. The decision of the multidisciplinary meeting was to propose carboplatin, paclitaxel, plus bevacizumab adjuvant chemotherapy with partial response at 3 months.

Patient received genetic counseling because of family history of cancers, and results returned positive in April 2017 showing a deleterious *BRCA1* germline mutation (*BRCA1* p.His1006Glnfs*17.c.3018_3021delTTCA) that motivated the use of PARPi. Olaparib was started (800 mg twice daily) in maintenance associated with bevacizumab. Somatic tumor testing showed that the BRCA1 germline mutation was associated with loss of heterozygosity and with a TP53 mutation p.Arg248Gln; c.743 G>A validating homologous recombination deficiency (HRD) in this tumor. The treatment was well tolerated, despite nausea, grade 1 asthenia, and grade 4 anemia leading to dose reduction (400 mg twice daily).

**Fig. 1:**
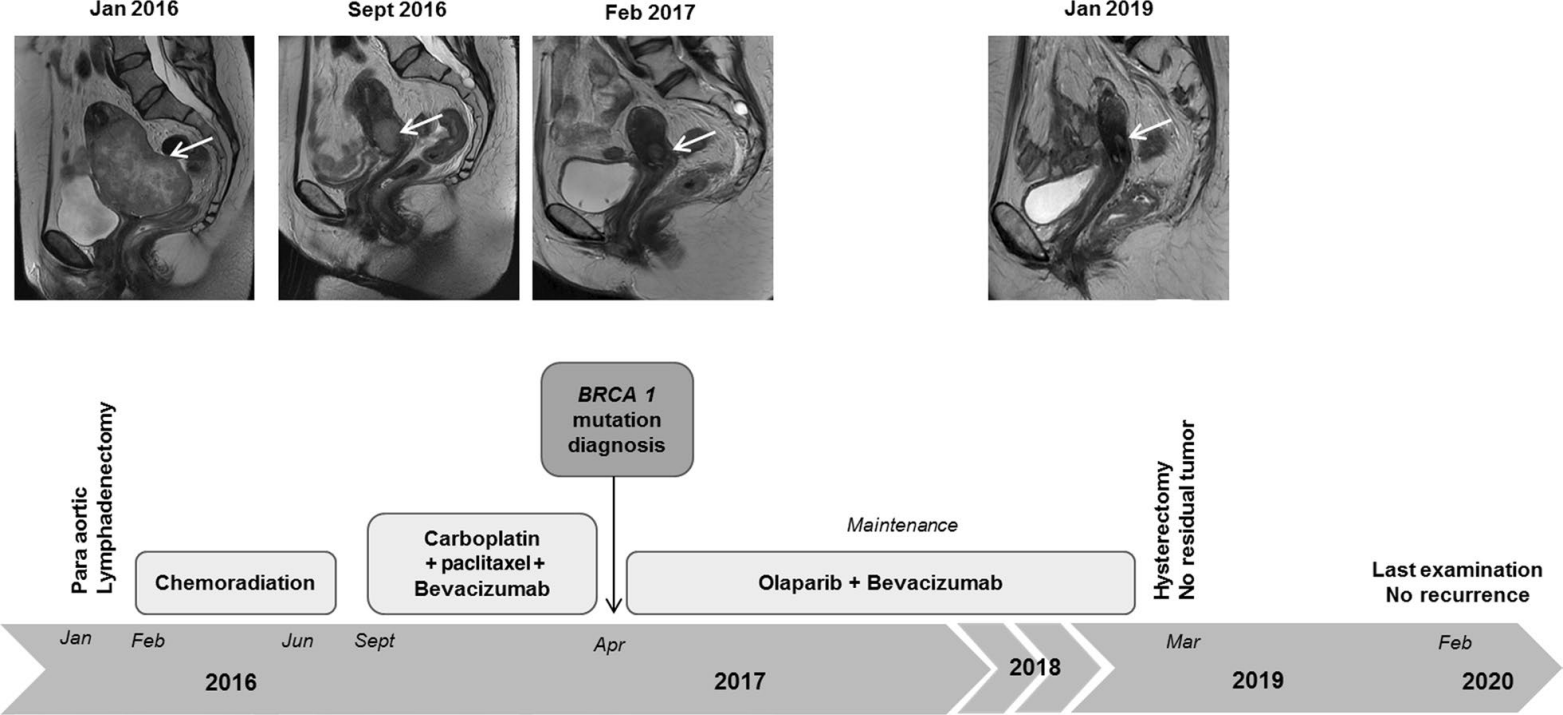
Timeline of treatment received and corresponding pelvic magnetic resonance imaging. The Arrow is pointing the cervix tumoral mass

In January 2019, pelvis MRI showed a decrease of nearly 50% in size of the tumor residue compared with previous examinations, with persistence of a left proximal infiltration of the parametrium and fibrous retraction of the left ureter. A timeline of treatment received and corresponding pelvic magnetic resonance imaging is shown in Fig. 1.

There was no argument on PET/CT for distant disease, and clinical benefit was reported.

The multidisciplinary meeting (multidisciplinary tumor board) discussed the option of surgery at that time. After reevaluation of patient’s medical record, we proposed surgery. A simple hysterectomy was performed in March 2019 without ureteral resection as parametrium appeared normal. No intra- or postoperative complications were noticed. Histological results showed no residual malignancy. After 1 year follow-up, clinical and radiological examinations do not show any recurrence without maintenance therapy.

## Discussion and conclusions

We report the first case of a *BRCA1* mutated patient with persistent advanced CC following chemoradiation and chemotherapy showing a complete tumor response after olaparib/bevacizumab adjuvant treatment and 12 months disease-free survival after surgery. The understanding of the molecular changes involved in the development of cancer led to the development of a new anticancer therapy known as targeted therapy and to a more personalized management of patients. In CC, therapies targeting different molecular pathways are investigated, including epidermal growth factor receptor (EGFR), vascular endothelial growth factor (VEGF), mammalian target of rapamycin (mTOR), and poly(ADP-ribose) polymerase (PARP). An analysis of 592 samples of cervical cancer in a tumor library in the US using a combination of sequencing (that is, next-generation sequencing), gene amplification (that is, *in situ* hybridization), and protein expression (that is, immunohistochemistry) identified mutations in 224 specimens (BRCA1 in 10%). These biomarkers could help guide therapy in clinical trials for patients with PARPi; mitogen-activated protein kinase, cell cycle checkpoint, and PI3K/AKT/mTOR pathway inhibitors; EGFR- and HER2-directed therapy; immunotherapy; and hormonal therapy, mainly in patients who have progressed with bevacizumab [11].

PARPi are being tested in different cancer trials. PARP are enzymes involved in different DNA repair pathways, most notably in the base excision repair pathway (BER) to repair single DNA strand breaks (SSBs). PARP inhibitors (PARPi) block PARP activity, resulting in increased DNA damage. Cells with HRD depend on *PARP*-mediated repair for survival. The dual blockade repairs pathways when PARPi are used in a background of HRD, resulting in synthetic lethality and cell death. The *BRCA* genes (*BRCA1 and BRCA2*) are major players of the HR repair pathway, and mutations in both genes predict response to PARPi in different cancer types [8, 9]. Currently, olaparib, rucaparib, and niraparib have been approved by the FDA and/or European Medicines Agency (EMA) for the treatment of ovarian cancer, while veliparib is in the late stage of clinical development. Talazoparib has been approved by the FDA for the treatment of metastatic germline BRCA1/2 mutated breast cancers in October 2018. The discovery and characterization of talazoparib as a potent, PARP1/2 inhibitor provides an important addition to the field of PARP inhibitors. Its potency in PARP trapping is early evidence that talazoparib could potentially lead to improved clinical outcomes in BRCA mutant malignancies [12].

PARP-1 is overexpressed in cancer cells, and higher expression of PARP-1 has been associated with chemoresistance [13]; other studies suggest that PARPi could sensitize cancer cells to chemotherapy [14]. *In vitro*, PARPi increase apoptosis in the HeLa CC cell line and sensitize cancer cells to cisplatin [15]. Prasad *et al.* showed that CC cells exhibit high amounts of PARP1 and that expression level is significantly higher in stages IIB and IIIB as compared with IIA (FIGO 2009) [16]. In this study, authors have proven that the replication stress caused by cisplatin induces PARP1 expression in a dose-dependent manner and determined the effect of olaparib on PARP1 activity in a PARP1 cisplatin-induced model. They showed that PARP1 inhibition enhanced cisplatin-induced DNA damage and apoptosis in CC cells and had a suppressive effect on metastasis. Altogether, olaparib increased cisplatin-induced lethality in CC cells. The authors hypothesized that combination of olaparib with cisplatin could enhance the efficacy of cisplatin in CC and improve the results of platinum-based therapy, and we believe that this hypothesis can explain the complete pathological response observed in our report, especially in the case of *BRCA* mutation.

Bianchi *et al.* explored the preclinical activity of olaparib *in vitro* and *in vivo* against nine primary CC cells [17]. PARP1 enzyme plays a key role in the recruitment of DNA repair factors and is also involved in the PARylation of nuclear proteins, a posttranslational modification process by which polymers of ADP-ribose (poly(adenosine diphosphate-ribose)) are covalently attached to proteins by PAR polymerase enzymes [18]. In this study, none of the cell lines demonstrated HRD but 33% showed strong PARylation activity associated with high sensitivity to the PARPi olaparib *in vitro*. In this subset of CC primary cells, olaparib significantly impaired CC xenograft tumor growth (*p* = 0.0017) and increased animal overall survival (*p* = 0.008). Authors suggest the use of the level of PARylation as a biomarker to predict for which patients PARPi would be the most efficient. PARP expression/activity is suggested to be mediated by chronic inflammation and HPV infection through oxidative stress that may result in DNA single-strand breaks [19–21]. HPV infection may promote progression by creating a vicious circle of inflammation and PARP activation. Thereby, PARPi may limit the role of PARP and consequently CC progression, whatever the *BRCA* or HRD status.

At a clinical level, the majority of data on PARPi in gynecological malignancies has been specifically focused on ovarian cancer, but PARPi are also under evaluation in the treatment of cervical and uterine cancers [9]. In CC, data from a phase I/II study assessing veliparib with cisplatin and paclitaxel in the treatment of advanced, recurrent, or persistent CC showed a promising overall response rate [22]. In this trial conducted by the Gynecologic Oncology Group including 34 patients, an overall response rate of 34% was observed for all dose levels and 60% for the maximum dose level. These results were not observed in another phase I study using veliparib and topotecan, which reinforces the idea of a synergic effect of PARPi and cisplatin [14]. A phase II randomized placebo-controlled double-blind study started in October 2019 to evaluate the efficacy and safety of rucaparib, another PARPi, as adjuvant treatment for patients with locally advanced CC who are responding to chemoradiation (ClinicalTrials.gov identifier: NCT03795272).

In conclusion, PARPi could be an alternative maintenance treatment for patients with persistent advanced CC previously treated with platinum. More studies are necessary to confirm the association between HRD in CC and response to PARPi, but it opens the way for personalized treatments in this type of cancer, especially when familial history of cancers is reported.

## Data Availability

The patient consent is available upon request.

## Abbreviations

CC: Cervical cancer
PARP: Poly(ADP-ribose) polymerase
PARPi: Poly(ADP-ribose) polymerase inhibitors
HRD: Homologous recombination deficiency
MRI: Magnetic resonance imaging
PET/CT: Positron emission tomography/computed tomography
HPV: Human papilloma virus
